# Attitudes and practices regarding preoperative psychological evaluation in orthognathic surgery: a scenario-based national survey of Turkish oral and maxillofacial surgeons

**DOI:** 10.1186/s12903-026-08928-w

**Published:** 2026-06-23

**Authors:** Şeydanur Urhan Güçlü, Pelin Aydın, Murat Kaan Erdem, Mustafa Ercüment Önder, Reha Ş. Kişnişci

**Affiliations:** 1 Department of Oral and Maxillofacial Surgery, Faculty of Dentistry, Lokman Hekim University, Söğütözü, 9 Eylül Street, Ankara, Çankaya 6 06510 Turkey; 2 Cleft Lip & Palate and Related Anomalies Center (DUDAMER), Ankara, Turkey

**Keywords:** Orthognathic surgery, Preoperative Care, Psychological tests, Decision making, Maxillofacial Surgery, Surveys and Questionnaires

## Abstract

**Background:**

Orthognathic surgery is known to influence patients’ psychological status and quality of life. While psychosocial outcomes have been widely investigated, limited data exist regarding oral and maxillofacial surgeons’ attitudes and clinical practices related to preoperative psychological evaluation. Understanding these perspectives is important, as unrecognized psychological concerns may affect treatment adherence, outcomes, and patient satisfaction. This study aimed to assess surgeons’ perspectives and reported practices regarding preoperative psychological evaluation in orthognathic surgery candidates and to identify any attitude–practice gap. Secondary aims were to evaluate variations across patient profiles using scenario-based assessments and to identify barriers influencing referral decisions.

**Methods:**

A cross-sectional, scenario-based online survey was conducted among oral and maxillofacial surgeons in Türkiye. The questionnaire included demographic items and scenario-based questions representing different clinical and psychosocial conditions, assessing both the perceived necessity and actual implementation of preoperative psychological evaluation. Descriptive statistics were used. Associations between categorical variables were analyzed using chi-square or Fisher’s exact tests. Differences between perceived necessity and actual practice were evaluated using the Wilcoxon signed-rank test. Scenario-based comparisons were performed using the Friedman test with Bonferroni-adjusted post hoc analyses where appropriate. A p-value < 0.05 was considered statistically significant.

**Results:**

Fifty-nine surgeons participated. Although most respondents (81.4%) considered preoperative psychological evaluation necessary, only 35.6% reported routinely conducting evaluations or referring patients. Among specific clinical scenarios, the highest level of support for preoperative psychological evaluation was observed in patients requesting gender transition (91.5%), although the survey did not distinguish supportive psychosocial assessment from mandatory psychiatric approval or clearance processes in these cases. High levels of support were also observed for patients presenting with unrealistic expectations (91.5%) or body dysmorphic features (86.4%), whereas support was lowest for patients with obstructive sleep apnea (27.1%) and older patients (49.2%). Perceived necessity varied significantly according to medical and surgical history (*p* < 0.001) but not by deformity type (*p* = 0.708). The most common barriers were anticipated negative patient reactions (40.7%) and limited knowledge of standardized tools (16.9%).

**Conclusions:**

Despite broad recognition of its importance, routine psychological evaluation remains inconsistently implemented in orthognathic surgery practice. Surgeons appeared more likely to support psychological referral in scenarios involving prominent psychosocial or identity-related concerns, whereas psychosocial considerations were less consistently emphasized in functionally indicated cases. These findings highlight the context-dependent nature of psychological referral tendencies and underscore the importance of context-sensitive multidisciplinary approaches in preoperative orthognathic care.

**Supplementary Information:**

The online version contains supplementary material available at https://doi.org/10.1186/s12903-026-08928-w.

## Introduction

Orthognathic surgery, which is performed to correct severe jaw misalignments, has a significant positive impact on both the aesthetic and functional aspects of patients’ lives. Studies consistently demonstrate that patients experience substantial improvements in facial appearance, with high rates of aesthetic satisfaction [1, 2]. Functionally, patients report enhanced chewing, speech, and occlusal function, with objective measures confirming improvements in bite alignment and facial balance [3, 4]. These changes are closely linked to enhanced psychological well-being and increased social confidence. Quality of life generally improves after surgery, with many patients’ post-treatment well-being approaching that of individuals without dentofacial deformities [3, 5].

Despite high satisfaction rates, a variety of patients remain dissatisfied with the treatment, regardless of its musculoskeletal successful outcome. This dissatisfaction is believed to be related to the patients’ expectations from orthognathic surgery, as well as their preoperative psychological state [6, 7]. Patients with dentofacial deformities, irrespective of the deformity’s severity, are known to experience psychological issues—such as depression, body dysmorphic disorder (BDD), and anxiety—at higher rates than the general population [8].

Although the impact of orthognathic surgery on patients’ quality of life and psychological status has been widely studied, there is a lack of research exploring surgeons’ routine clinical practices towards the preoperative psychological evaluation [3, 9, 10]. Evidence specifically focusing on oral and maxillofacial surgeons (OMFSs) remains limited, particularly regarding how psychological referral decisions vary across different clinical scenarios. The primary aim of this study was to assess the perspectives and reported practices of OMFSs regarding the use of preoperative psychological evaluation in candidates for orthognathic surgery, with a particular focus on identifying the presence and extent of any attitude–practice gap in this domain. The secondary aims were: (1) to examine how surgeons’ support for psychological evaluation varies across specific patient profiles using context-sensitive clinical scenarios; and (2) to identify perceived barriers and facilitating factors (such as patient willingness, time constraints, and the quality of referral pathways) that influence surgeons’ decision-making related to psychological referral. Unlike previous studies focusing primarily on general attitudes toward psychological assessment, the present study was designed as an exploratory survey incorporating scenario-based clinical evaluations to better understand how psychological referral tendencies vary across different orthognathic patient contexts within an OMFSs population.

## Materials and methods

This exploratory cross-sectional, scenario-based online survey was conducted using a self-administered questionnaire developed in Google Forms to investigate the attitudes and practices of OMFSs regarding preoperative psychological evaluation in orthognathic surgery patients. The questionnaire included original items and modified items adapted from earlier studies [10–12]. The questionnaire link was distributed via e-mail to all registered members of the Association of Oral and Maxillofacial Surgery Society (ACBİD) in June 2025.

The survey consisted of 5 main sections:

1. Sociodemographic characteristics (e.g., age, gender, institution type, years of clinical experience).
2. Clinical workload and experience with orthognathic surgery.
3. Current practices in psychological evaluation (e.g., frequency, referral habits, tools used).
4. Perspectives on psychosocial risk factors and scenario-based indications for psychological referral in orthognathic surgery candidates.
5. Perceived barriers and facilitators related to the implementation of psychological assessment in surgical planning.

Scenario-based items explored surgeons’ perceptions regarding the necessity of psychological or psychiatric referral across different clinical and psychosocial contexts relevant to orthognathic and related facial surgical care, including revision surgery, cleft-related deformities, obstructive sleep apnea, and patients undergoing gender transition. No further distinction was made between conventional orthognathic procedures and broader gender-affirming facial procedures.

The questionnaire also included multiple items assessing perceived barriers to preoperative psychological evaluation. For inferential statistical analysis, conceptually related items were consolidated into five broader thematic categories: patient-related concerns, clinical beliefs and institutional policies, lack of knowledge regarding assessment tools, referral network limitations, and logistical or financial constraints.

All questionnaire items were structured as single-choice questions, requiring participants to select a single response option for each item; multiple responses were not permitted. The complete questionnaire is available as Supplementary File 1. Participation was fully voluntary and anonymous, and respondents could not be identified or recontacted. Only responses submitted between June 1 and July 1, 2025, were included in the analysis.

Statistical analyses were performed using IBM SPSS Statistics for Windows, Version 29.0 (IBM Corp., Armonk, NY, USA). Descriptive statistics, including mean, standard deviation, median, frequency, and percentage, were used to summarize the data. Normality of continuous variables was assessed using the Shapiro–Wilk test, which demonstrated non-normal distributions (*p* < 0.001). Due to the ordinal nature of Likert-scale data, the categorical structure of several variables, and the non-normal distribution of multiple measures, non-parametric statistical methods were used throughout the analyses. The Wilcoxon signed-rank test was used to compare paired ordinal variables (perceived benefit and actual referral rates). The Friedman test was employed to compare multiple related Likert-scale items across clinical indications and scenarios; where significant, post-hoc pairwise comparisons were conducted using Wilcoxon signed-rank tests with Bonferroni correction (adjusted α = 0.05/55 = 0.0009). Relationships between categorical variables were analyzed using the chi-square test, with Fisher’s exact test applied where expected cell counts were less than five. A goodness-of-fit chi-square test was used to assess the distribution of barrier categories. Statistical significance was set at *p* < 0.05.

This cross-sectional survey study was approved by the institutional review board of Lokman Hekim University (Protocol number 2025066).

The study is reported in accordance with the CHERRIES (Checklist for Reporting Results of Internet E-Surveys) guidelines. A completed checklist is provided as a supplementary file (Supplementary file 2).

## Results

A total of 59 OMFSs participated in the study within the specified time frame, representing an overall response rate of 8.4%. Among them, 24 were male (40.7%), and the mean age was 33.81 ± 7.64 years. Regarding professional status, 35.6% were faculty members, 33.9% were currently in specialty or doctoral training, and 30.5% had completed their training. Most participants (89.8%) were working in university or training hospitals. In terms of clinical exposure, 47.5% reported evaluating 1–10 orthognathic surgery patients annually (Table 1).

**Table 1:** Demographic characteristics of the participants

Variable	Subgroups	*n* (%)
Gender	Female	35 (59,3%)
	Male Total	24 (40,7%) 59 (100%)
Age (years)	Mean ± SD	33.81 ± 7.64
	Minimum–Maximum	24–55
Level of Training	Currently in residency/PhD training	20 (33,9%)
	Completed residency/PhD training	18 (30,5%)
	Trainer/faculty member	21 (35,6%)
Institution Type	University / training hospital	53 (89,8%)
	Private hospital	2 (3,4%)
	Private clinic	2 (3,4%)
	Public hospital	1 (1,7%)
	Oral and dental health center	1 (1,7%)
Years of Professional Experience	0–5 years	24 (40,7%)
	6–10 years	17 (28,8%)
	More than 10 years	18 (30,5%)
Number of Orthognathic Patients Evaluated in the Last Year	1–10 patients	28 (47,5%)
	11–25 patients	18 (30,5%)
	26–50 patients	9 (15,3%)
	51 or more patients	4 (6,8%)

In terms of preoperative psychological assessment, 64.4% of the respondents stated that they did not routinely conduct psychological evaluations in their patients undergoing orthognathic surgery. Among those who reported performing such evaluations (*n* = 21), 61.9% relied primarily on interviews based on personal clinical impressions, 38.1% referred patients to a psychologist or psychiatrist, and one participant indicated using a specially developed scale without specifying its name.

Regarding the perceived necessity of psychological evaluation, 81.4% (*n* = 48) of respondents stated that preoperative psychological assessment should be routinely performed for all orthognathic surgery patients. Conversely, 8.5% (*n* = 5) disagreed, and 10.2% (*n* = 6) were undecided.

**Fig. 1:**
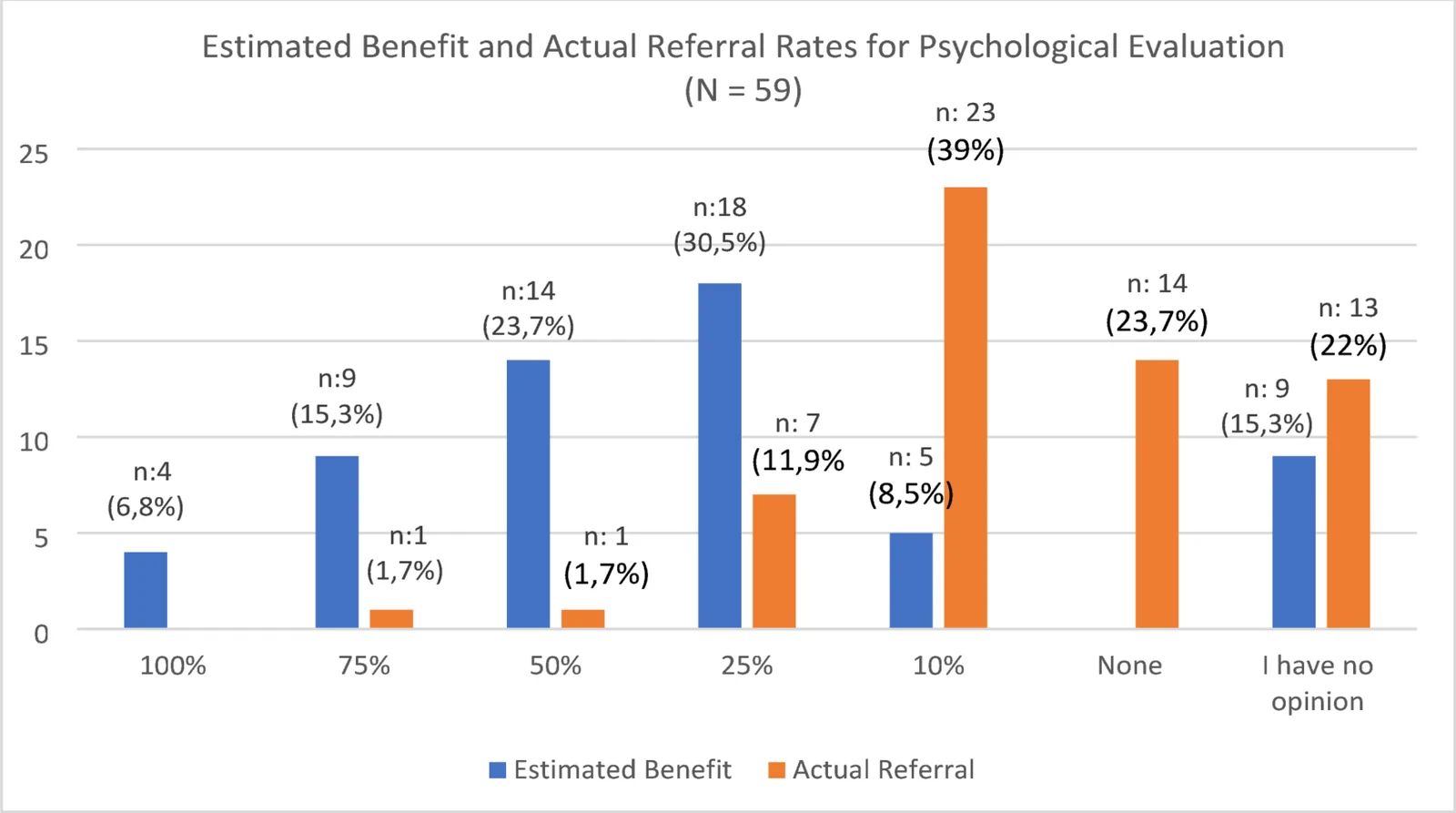
The discrepancy between estimated clinical benefit and actual referral frequency in orthognathic surgery. The bar chart illustrates the distribution of surgeons’ estimates regarding the proportion of orthognathic surgery patients who could benefit from psychological referral and the proportion actually referred. While 45.8% of respondents estimated that at least half of their patients could benefit from psychological referral, only 3.4% reported referring patients at this frequency. Actual referral responses were concentrated in the 10%, none, and “I have no opinion” categories, highlighting a discrepancy between perceived benefit and routine clinical implementation

Participants were asked to estimate the proportion of their orthognathic surgery patients who could potentially benefit from psychological or psychiatric referral, and the proportion who were actually referred. The full distribution of responses is presented in Fig. 1. While 72.9% of respondents (*n* = 43) believed that at least 25% of their patients could benefit from referral, only 16.9% (*n* = 10) reported actually referring patients at this level. Respondents who selected “I have no opinion” for either or both estimation items were excluded via listwise deletion (perceived benefit estimate only: *n* = 5; actual referral rate only: *n* = 9; both items: *n* = 4), yielding a valid analytic sample of 41. Excluded respondents were more likely to be early-career surgeons (0–5 years of experience: 61.1% vs. 31.7%), currently in training (55.6% vs. 24.4%), and to report lower case volumes (1–10 cases/year: 72.2% vs. 36.6%) compared with the analytic sample (Supplementary file 3). A Wilcoxon signed-rank test revealed a statistically significant discrepancy between perceived benefit and actual referral rates (T = 8.5, Z = − 5.17, *p* < 0.001), with a large effect size (*r* = 0.807, calculated as Z/√N, *N* = 41), confirming a substantial attitude–practice gap in clinical implementation.

In regards about the main barriers preventing them from conducting or referring patients for preoperative psychological evaluation. Barriers were categorized into five groups. A goodness-of-fit chi-square test indicated that responses were not uniformly distributed across barrier categories (χ²(4) = 18.71, *p* < 0.001). Patient-related concerns were the most frequently cited barrier (*n* = 24, 40.7). Other significant barriers included clinical beliefs and institutional policies (*n* = 12, 20.3%), and a lack of knowledge regarding reliable assessment tools (*n* = 10, 16.9%) (Table 2).

**Table 2:** Barriers preventing surgeons from performing or referring preoperative psychological evaluations

Barrier Category (Theme)	*n* (%)
Patient-Related Concerns (Fear of negative reaction or refusal)	24 (40.7%)
Clinical Beliefs and Institutional Policy (Not routinely necessary, preference for post-op, department policy)	12 (20.3%)
Lack of Knowledge and Tools (Unfamiliarity with reliable scales/questionnaires)	10 (16.9%)
Referral Network Issues (Not knowing a specialist or uncertainty about whom to refer)	9 (15.3%)
Logistical and Financial Constraints (Time constraints and cost of referral)	4 (6.8%)
Total	59 (100%)

Participants rated the necessity of psychological referral across 11 clinical indications using a 5-point Likert scale. A Friedman test revealed a statistically significant difference in the perceived necessity of psychological referral across clinical indications (χ²(10) = 150.24, *p* < 0.001), indicating a moderate level of agreement (Kendall’s W = 0.25).

The highest mean ranks were observed for suspected BDD (Mean Rank = 8.01) and unrealistic patient expectations (Mean Rank = 7.50), with a high level of agreement among participants (≥ 4 responses: 90% and 92%, respectively). In contrast, lower mean ranks were observed for patients seeking multiple medical opinions (Mean Rank = 3.31) and those presenting with recent anxiety (Mean Rank = 4.36), which were associated with lower agreement (Table 3). Post-hoc pairwise comparisons using Wilcoxon signed-rank tests with Bonferroni correction (adjusted α = 0.0009) revealed that high-priority indications were rated significantly higher than lower-priority items (*p* < 0.001).

**Table 3:** Surgeons’ referral decisions for preoperative psychological evaluation in various clinical scenarios

Clinical Scenario	Mean Score	SD	% ≥4	Mean Rank
Suspected body dysmorphic disorder	4.53	0.728	89,8	8.01
Unrealistic patient expectations	4.39	0.695	91.6	7.50
Surgery requested despite minimal functional/aesthetic issues	4.31	0.623	94.9	7.03
Current or past psychiatric history	4.22	0.789	84.8	6.88
Surgeon’s clinical impression of psychological risk	4.22	0.494	96.6	6.67
Inadequate coping with perioperative stress	4.03	0.718	86.4	6.10
Significant social anxiety or low self-esteem	3.86	0.840	74.5	5.48
Family pressure or objection to surgery	3.85	0.867	71.2	5.32
Communication or compliance problems during treatment	3.80	0.83	78.0	5.33
Recent anxiety specifically about the surgery	3.54	0.816	57.7	4.36
Seeking multiple medical opinions	3.15	1.031	42.4	3.31

Values are presented as mean ± standard deviation (SD), percentage of participants rating the item ≥ 4 on a 5-point Likert scale, and mean ranks derived from Friedman test analysis. Higher mean ranks indicate greater perceived importance of the indication for psychological referral

The perceived necessity of preoperative psychological evaluation was further assessed across 11 distinct clinical scenarios, categorized into demographic variables, deformity and surgery types, and medical/surgical history factors. A Friedman test demonstrated significant differences across scenarios (χ²(10) = 184.54, *p* < 0.001), indicating a moderate level of agreement among participants (Kendall’s W = 0.31).

The strongest consensus was observed within the medical and surgical history subgroup (χ²(3) = 95.50, *p* < 0.001; Kendall’s W = 0.54), indicating a large effect size. Mean rank analysis showed a clear prioritization pattern, with gender transition and revision surgery rated highest, and obstructive sleep apnea consistently rated lowest (Table 4).

**Table 4:** Perceived necessity of preoperative psychological evaluation across patient scenarios

Patient Group	Mean Score	SD	% ≥4	Mean Rank
a. Demographic Factors				
a1. Patients with ongoing growth	3.49	0.989	59.4	6.03
a2. Young patients (16–25 years)	3.78	0.789	72.9	6.90
a3. Elderly patients	3.39	1.000	49.2	5.58
b. Deformity and Type of Surgery				
b1. Borderline skeletal Class III cases	3.32	1.136	49.2	5.45
b2. Genioplasty cases	3.22	0.911	39.0	5.20
b3. Skeletal Class III cases	3.08	1.103	39.0	4.60
b4. Skeletal Class II cases	3.14	1.008	39.0	4.73
c. Medical and Surgical History Factors				
c1. Patients with obstructive sleep apnea	2.80	0.961	27.1	3.61
c2. Patients in gender transition	4.53	0.704	91.5	9.26
c3. Revision after previous orthognathic/aesthetic surgery	4.05	0.860	76.3	7.86
c4. Patients with cleft lip/palate	3.71	1.115	66.1	6.79

Table values are presented as mean ± standard deviation (SD), percentage of participants rating the necessity as ≥ 4 on a 5-point Likert scale, and mean ranks derived from Friedman test analysis. Higher mean ranks indicate greater perceived necessity of preoperative psychological evaluation across patient scenarios. Friedman test results by subgroup were as follows: demographic factors, χ²(2) = 6.14, *p* = 0.046, Kendall’s W = 0.05; deformity and type of surgery, χ²(3) = 5.59, *p* = 0.134, Kendall’s W = 0.03; medical and surgical history factors, χ²(3) = 95.50, *p* < 0.001, Kendall’s W = 0.54

Within the demographic subgroup, a borderline statistically significant difference was observed (χ²(2) = 6.14, *p* = 0.046); however, the associated effect size was negligible (Kendall’s W = 0.05), suggesting limited clinical relevance. No statistically significant differences were identified within deformity and surgery type categories (χ²(3) = 5.59, *p* = 0.134), and the associated effect size was negligible (Kendall’s W = 0.03) (Table 4).

Notably, statistical analysis revealed no significant association between reporting any specific barrier and variables such as workplace, years of experience, or annual orthognathic patient volume (*p* > 0.05 for all comparisons, Fisher’s exact test). Similarly, the belief that psychological evaluation should be mandatory for all orthognathic patients was not significantly associated with the surgeon’s level of training or their current routine of performing psychological assessments (*p* > 0.05, Fisher’s exact test).

## Discussion

Beyond conventional functional and esthetic outcome measures, patient-reported quality of life and personal perceptions play a critical role in evaluating treatment success following orthognathic surgery. Quality of life encompasses multiple domains, including physical health, psychological well-being, social functioning, and environmental factors [13]. Condition-specific assessment tools further emphasize the multidimensional nature of surgical outcomes, particularly in relation to facial aesthetics, oral function, social interactions, and awareness of dentofacial deformity [14].

Patients with dentofacial deformities frequently exhibit reduced self-esteem and elevated levels of anxiety and depression compared with the general population, contributing to psychosocial impairment that extends beyond physical limitations [8, 15]. In this context, surgical correction alone may not guarantee postoperative satisfaction, especially in patients with underlying psychological vulnerability or unrealistic expectations regarding treatment outcomes.

Accordingly, preoperative psychological assessment, once considered unnecessary in routine practice, is now increasingly recognized as integral to orthognathic care [16, 17]. Previous studies have demonstrated its value in identifying at-risk patients, facilitating expectation management, improving emotional preparedness, and enhancing overall patient satisfaction [7, 8, 18]. Despite this growing recognition, the perspectives and implementation practices of OMFSs regarding psychological evaluation remain insufficiently explored, particularly in relation to context-specific clinical decision-making. The present study contributes to this relatively limited area of research by providing preliminary data on OMFS’ attitudes, perceived indications, implementation practices, and scenario-based referral tendencies related to preoperative psychological assessment. While prior studies in this domain have largely sampled orthodontists or mixed orthodontic–surgical populations, the present study specifically targets OMFSs — the clinicians who ultimately perform the procedure and bear primary responsibility for surgical decision-making and patient selection. Surgeons’ referral behavior and perceived barriers may differ meaningfully from those of orthodontists, given their distinct clinical roles, training backgrounds, and patient interaction patterns.

Despite widespread professional acknowledgment of the importance of preoperative psychological assessment, its translation into routine clinical practice remains inconsistent, and highlights a pronounced attitude–practice gap. A further discordance is evident between surgeons’ perceived benefit estimates and their actual referral behavior — a pattern observed across multiple surgical disciplines and consistent with findings from the broader implementation science literature [19–21]. Consequently, psychologically vulnerable individuals may remain unidentified throughout the treatment process, increasing the risk of unmet expectations, postoperative dissatisfaction, and suboptimal psychosocial adaptation.

The most frequently reported barrier to psychological referral in this study was surgeons’ concern that patients might react negatively or refuse assessment. This pattern parallels findings from earlier literature, suggesting that apprehension about damaging the therapeutic relationship remains a persistent influence on clinical decision-making [11]. Notably, most studies documenting this pattern have been conducted in Western European or Anglo-American settings. This barrier has also been identified in a Turkish orthodontic cohort, where fear of patient refusal was similarly reported as a primary reason for not referring patients to psychological support [12]. The present study extends these findings to an oral and maxillofacial surgery population, representing the first investigation of this barrier specifically within a Turkish OMFSs context. Such concerns may also reflect broader sociocultural attitudes toward mental health and psychiatric referral, particularly in settings where mental health consultation may still be associated with stigma, social labeling, or misconceptions regarding psychiatric illness. Previous studies conducted in Türkiye have demonstrated that individuals with mental health conditions frequently experience stigmatization, social withdrawal, concealment of illness, and fear of negative societal attitudes [22]. These perceptions may indirectly shape surgeons’ expectations regarding patients’ responses to psychological referral. In addition, cross-cultural mental health literature emphasizes that stigma surrounding psychiatric care remains a persistent barrier across healthcare systems and highlights the importance of culturally sensitive anti-stigma approaches [23, 24]. Notably, recent patient-focused studies indicate that when the purpose of psychological evaluation is clearly explained, patients report high acceptance and satisfaction, challenging the perception that resistance is inevitable [18]. These findings underscore the importance of clear and supportive communication strategies to mitigate anticipated negative reactions and facilitate appropriate referrals.

Additional system-level factors also appeared to limit the routine use of psychological assessment. Many surgeons reported being unfamiliar with appropriate screening instruments—a notable finding given the wide range of validated tools available in orthognathic research [7, 9, 14, 17]. This lack of familiarity may reflect gaps in specialty training, but it may also stem from the heterogeneity of existing measures, which vary widely in purpose, structure, and clinical applicability. The coexistence of numerous instruments, coupled with the absence of a single, widely endorsed screening tool tailored to orthognathic surgery, likely contributes to uncertainty about which measure should be used in everyday clinical practice. A similar ambiguity was reflected in surgeons’ reports of not knowing which mental health professional to refer patients to, suggesting that gaps in training are compounded by the lack of structured, easily accessible referral pathways. The higher proportion of “No opinion” responses among early-career and lower-volume surgeons may also suggest greater uncertainty regarding psychosocial referral practices within less experienced groups. Addressing these challenges will require clearer guidance on practical, evidence-based screening options, alongside institutionally supported referral networks that facilitate timely collaboration with mental health specialists.

The practical consequence of these systemic challenges is most evident in the specific methods used for patient evaluation. Although numerous psychometric instruments are available for evaluating psychological vulnerability in orthognathic surgery patients, these tools remain largely underutilized in routine practice [3, 16, 19]. Our findings indicate that assessment is predominantly based on unstructured clinical impressions and subjective judgment rather than standardized criteria. Only a single respondent reported using a dedicated screening scale, without specifying the instrument employed, underscoring the limited integration of objective assessment methods.

The low utilization of psychometric tools may reflect practical barriers, including time constraints, limited training in psychological assessment, and uncertainty regarding the clinical applicability of existing instruments [25]. In addition, restricted access to mental health professionals and the absence of established multidisciplinary referral pathways further compound reliance on informal evaluation. Consequently, the lack of a simple, validated, surgery-specific screening instrument for preliminary patient triage represents a critical gap, particularly for clinics where routine psychiatric consultation is not readily available. The development of standardized assessment tools may therefore reduce subjective variability and support more consistent identification of patients requiring psychosocial care.

Participants showed strong agreement regarding the primary indicators for psychological referral, particularly in cases involving clear red-flag features such as body dysmorphic concerns or unrealistic expectations—findings consistent with existing evidence linking these presentations to postoperative dissatisfaction and maladaptive psychosocial outcomes [19, 26]. However, this consensus did not extend to less overt but clinically relevant situations, such as patients seeking multiple medical opinions or presenting with elevated preoperative anxiety. The former may, in some cases, reflect uncertainty about treatment or difficulty maintaining realistic expectations—features discussed in the clinical literature on body image–related concerns even when diagnostic criteria are not met [19]. Likewise, heightened preoperative anxiety has been associated with poorer postoperative psychological adjustment, yet it was not consistently recognized as an indication for referral [16]. Together, these patterns suggest that psychological risk assessment tends to focus on clearly identifiable risk markers, whereas more subtle but clinically significant vulnerabilities may be underestimated.

The variation in surgeons’ responses across patient groups suggests that perceptions of psychological assessment are shaped by the clinical context. Strong support for evaluation in gender transition cases aligns with literature describing the complex psychosocial needs and heightened expectations often observed in this population [27]. However, this finding should be interpreted cautiously, particularly given the broad wording of the survey scenario, which referred to orthognathic surgery patients in the context of gender transition rather than gender-affirming facial surgery specifically. Participants may therefore have interpreted the scenario in different ways. One possible explanation is that surgeons perceived these patients as potentially benefiting from more comprehensive multidisciplinary psychosocial support. At the same time, contemporary gender-affirming care frameworks increasingly emphasize supportive and individualized assessment approaches rather than rigid gatekeeping-oriented psychiatric evaluation models, underscoring the importance of avoiding interpretations that may inadvertently pathologize gender identity [28].

By contrast, lower support for psychological assessment in patients with obstructive sleep apnea indicates that these cases may be viewed primarily in terms of their functional characteristics, even though chronic sleep disorders have been associated with emotional distress, reduced quality of life, and psychological morbidity [29]. The greater uncertainty observed in revision surgery patients may similarly reflect the challenge of distinguishing ongoing functional concerns from underlying psychosocial distress, heightened expectations, or body image–related difficulties [30, 31]. Taken together, these findings suggest that surgeons may be more attuned to psychological needs when overt aesthetic or identity-related factors are present, whereas patients whose primary indications appear functional may receive less consistent psychosocial consideration.

Participants’ responses regarding demographic factors showed only borderline statistical significance with a negligible effect size. However, given the modest sample size, the possibility of Type II error cannot be excluded. Nevertheless, the findings suggest that surgeons may maintain a largely age-neutral approach when considering the need for preoperative psychological evaluation. From a clinical perspective, this broad, age-neutral approach may represent a strength, as psychological vulnerability is known to manifest differently across the lifespan rather than being confined to a single demographic group. The literature describes distinct psychosocial considerations in younger patients, including ongoing identity formation and heightened sensitivity to appearance changes [32], while older adults may face increased emotional burden associated with chronic medical conditions, functional limitations, or reduced social support [33, 34]. For patients still undergoing craniofacial growth, the psychosocial dynamics of both the patient and the family must also be considered [35]. Taken together, the absence of age-based differences in surgeons’ responses underscores an encouraging willingness to consider psychological assessment across the full orthognathic patient spectrum.

Although no statistically significant differences were observed across deformity classifications or surgical procedure types, the possibility of Type II error cannot be excluded given the modest sample size and negligible observed effect size. Nevertheless, two descriptive trends merit further consideration. First, borderline Class III cases—often characterized by treatment planning ambiguity—received slightly higher priority ratings. While literature suggests skeletal Class III patients may face increased psychosocial risks [9, 36, 37], the specific ‘borderline’ subgroup remains under-researched. This observed trend may reflect heightened clinical caution in cases where decisional uncertainty necessitates more complex expectation management. Second, isolated genioplasty procedures elicited greater variability in responses, potentially due to the heterogeneous motivations for this surgery, ranging from purely aesthetic to functional concerns [38]. Collectively, these observations suggest that surgeons’ perceptions of psychosocial vulnerability may be guided more by patient-specific variables—such as individual expectations—than by rigid deformity classifications or procedural categories.

Notably, surgeons’ perceptions did not differ according to experience level or workplace setting. This consistency suggests that views on psychological assessment are shaped by shared professional norms and common institutional challenges rather than isolated individual factors. Such a common baseline provides an opportunity for field-wide initiatives—such as enhanced training and clearer referral pathways—to be implemented broadly and with potentially uniform impact across different practice environments. To our knowledge, this consistency across experience levels and workplace settings has not been systematically examined in prior orthognathic surgery literature, and may have particular relevance for the design of field-wide rather than individually targeted interventions.

Several limitations should be considered when interpreting the findings of this study. The overall response rate of 8.4% is notably low and warrants careful consideration beyond survey fatigue alone. While declining engagement with online surveys has been widely documented and attributed to competing professional demands [10, 12], a response rate at this level raises substantive questions about representativeness. The 91.6% of registered members who did not participate may differ systematically from respondents in ways directly relevant to the study outcomes — for example, high-volume orthognathic surgeons with established referral protocols may have been less motivated to engage with a survey on psychological evaluation, whereas clinicians with a pre-existing interest in psychosocial aspects of care may have been overrepresented. If so, the observed attitude–practice gap and the scenario ratings may reflect a more psychosocially oriented subgroup rather than the broader professional community. A further factor potentially inflating the observed gap is the listwise deletion of 18 respondents who were predominantly early-career surgeons with limited case volumes; as a result, the analytic sample was enriched for more experienced surgeons, and the magnitude of the attitude–practice gap should be interpreted with caution. As comparative demographic data for the full registered membership are not publicly available, a formal non-response analysis could not be conducted; accordingly, the findings should be interpreted as reflecting a self-selected sample of Turkish OMFSs responding to an online survey, rather than the profession as a whole. Therefore, the direction and magnitude of potential nonresponse bias remain uncertain. Future studies may benefit from incorporating nonresponse analyses or follow-up surveys of non-participants to better assess representativeness. In addition, a substantial proportion of respondents reported lower orthognathic surgical case volumes, which may further limit representativeness with respect to high-volume surgical centers. The exclusive reliance on self-reported data constitutes another important limitation, as such responses may be influenced by recall bias or social desirability bias and may not fully reflect actual clinical behaviors; moreover, no objective verification of referral practices was available. In addition, the scenario referring to patients in the gender transition process may have been interpreted variably by respondents, as no explicit distinction was made between orthognathic surgery and gender-affirming facial procedures. This ambiguity may have influenced participants’ responses and represents a limitation of the study. Furthermore, the electronic distribution method may have introduced selection bias by underrepresenting clinicians less active within online professional or academic platforms. In addition, all authors were affiliated with a single institution, which may have introduced institutional framing bias in questionnaire design and interpretation. Finally, as this study was conducted within a specific geographical context, the findings may reflect local healthcare structures and cultural attitudes toward mental health, which may differ internationally. Collectively, these factors necessitate cautious interpretation regarding the generalizability of the results.

Despite these limitations, the present study offers several important contributions to the orthognathic literature. By utilizing scenario-based clinical decision items, we evaluated referral attitudes within realistic contexts, providing a more nuanced and grounded understanding than traditional opinion-based surveys. This approach effectively captured the application of psychological considerations in everyday clinical reasoning, potentially reducing the social desirability bias inherent in direct questioning. Furthermore, assessing diverse patient profiles enabled a comparative analysis of referral patterns, identifying specific clinical gaps—such as the perceived lower risk in functional cases. Together, these methodological features establish a solid foundation for developing structured, evidence-based psychosocial assessment pathways tailored specifically for orthognathic surgery.

Building on these strengths and acknowledging the study’s limitations, future research should adopt more diverse methodological approaches to deepen understanding in this area. In particular, the scenario-based methodology employed in the present study may serve as a replicable framework for future investigations across different surgical specialties and cultural contexts, allowing for systematic cross-cultural and cross-specialty comparisons of referral decision-making. The patient profiles and clinical scenarios developed here could similarly be adapted and validated for use as structured educational tools in residency training programs, where exposure to psychosocial decision-making remains limited. Qualitative designs—such as in-depth interviews or focus groups—could explore how surgeons communicate psychological concerns, navigate referral decisions, and interpret patient reactions in real clinical settings. Prospective, multicenter studies are also needed to evaluate whether introducing standardized psychological screening tools influences surgical outcomes, patient satisfaction, expectation management, or postoperative psychosocial adjustment. Finally, targeted educational interventions focusing on psychosocial assessment and referral communication could be tested to determine their effectiveness in reducing the practical barriers identified in the present study. These directions would help translate the current findings into more refined, evidence-based models of psychological evaluation in orthognathic surgery.

## Conclusion

This study demonstrates that although most OMFSs recognize the importance of preoperative psychological assessment in orthognathic surgery, its routine application remains inconsistent in clinical practice. Several findings from this study suggest context-sensitive directions for improvement. As concerns about negative patient reactions emerged as the most frequently reported barrier, communication training focused on introducing psychological referral in a supportive and non-stigmatizing manner may be particularly relevant, especially in settings where mental health stigma may influence patient acceptance. In addition, the higher proportion of “No opinion” responses observed among early-career surgeons with limited case volumes may indicate greater uncertainty regarding psychosocial referral practices. Structured exposure to psychosocial assessment and referral processes during residency training may therefore help support clinical confidence in this area. Unfamiliarity with referral resources further highlights the potential value of clearer institutional referral pathways, particularly within university and training hospital settings. Finally, the lower support observed for psychological evaluation in functionally indicated cases such as obstructive sleep apnea suggests that psychosocial considerations may be applied inconsistently across different orthognathic surgery contexts. Collectively, these findings highlight the context-dependent nature of psychological referral practices and underscore the value of specialty-specific and culturally informed approaches to multidisciplinary orthognathic care. The scenario-based methodology employed in this study further demonstrates that surgeons’ psychosocial reasoning varies meaningfully across patient profiles — a pattern not easily captured through generalized attitude-based surveys.

## Supplementary Information

Supplementary Material 1.

Supplementary Material 2.

Supplementary Material 3.

## Data Availability

The datasets generated and/or analyzed during the current study are available from the corresponding author on reasonable request. All data have been de-identified prior to sharing.

## References

[1] Ghorbani F, Gheibollahi H, Tavanafar S, Eftekharian HR. Improvement of Esthetic, Functional, and Social Well-Being After Orthognathic Surgical Intervention: A Sampling of Postsurgical Patients Over a 10-Year Period From 2007 to 2017. J Oral Maxillofac Surg. 2018;76(11):2398–403. 10.1016/j.joms.2018.04.034. 29792834

[2] Baniulyte G, Esson M. Exploring patient perspectives: orthognathic surgery outcomes and experiences assessed by patient-reported outcome measures and patient-reported experience measures. Br J Oral Maxillofac Surg. 2024;62(8):722–8. 10.1016/j.bjoms.2024.06.009. 39127557

[3] Cremona M, Bister D, Sheriff M, Abela S. Quality-of-life improvement, psychosocial benefits, and patient satisfaction of patients undergoing orthognathic surgery: a summary of systematic reviews. Eur J Orthod. 2022;44(6):603–13. 10.1093/ejo/cjac015. 35511144

[4] Simões JCM, Garcia DM, De Mello-Filho FV, De Felício CM, Trawitzki LVV. Masticatory function and three-dimensional facial morphology of soft tissues: One year after orthognathic surgery. Arch Oral Biol. 2025;169:106103. 10.1016/j.archoralbio.2024.106103. 39426314

[5] Belusic Gobic M, Kralj M, Harmicar D, Cerovic R, Mady Maricic B, Spalj S. Dentofacial deformity and orthognatic surgery: Influence on self-esteem and aspects of quality of life. J Craniomaxillofac Surg. 2021;49(4):277–81. 10.1016/j.jcms.2021.01.024. 33579617

[6] Shi P, Huang Y, Kou H, Wang T, Chen H. Risk Factors for Facial Appearance Dissatisfaction Among Orthognathic Patients: Comparing Patients to a Non-Surgical Sample. Front Psychol. 2019;10:2775. 10.3389/fpsyg.2019.02775. 31920824 PMC6917591

[7] Huang YS, Chin WC, Yao CF, Chen YA, Tang I, Chen YR, et al. Sleep, Distressed Appearance, and Quality of Life Relate to Satisfaction with Orthognathic Surgery. Int J Environ Res Public Health. 2021;18(21):11253. 10.3390/ijerph182111253. 34769770 PMC8583211

[8] Wang BL, Yang ML. Psychological status of patients with skeletal Class III malocclusion undergoing bimaxillary surgery: A comparative study. Med (Baltim). 2024;103(34):e39435. 10.1097/MD.0000000000039435. PMC11346842 39183428

[9] Sun H, Shang HT, He LS, Ding MC, Su ZP, Shi YL. Assessing the Quality of Life in Patients With Dentofacial Deformities Before and After Orthognathic Surgery. J Oral Maxillofac Surg. 2018;76(10):2192–201. 10.1016/j.joms.2018.03.026. 29684310

[10] Sinnott PM, Hunt N, Shute J, Cunningham S. Psychological support for orthognathic patients: Who is doing what? J Orthod. 2020;47(3):205–12. 10.1177/1465312520929032. 32500802

[11] Juggins KJ, Feinmann C, Shute J, Cunningham SJ. Psychological support for orthognathic patients – what do orthodontists want? J Orthod. 2006;33(2):107–15. 10.1179/146531205225021492. 16751432

[12] Olkun HK. Orthodontists’ knowledge and experience on referring orthognathic surgery patients to psychological support. J Orofac Orthop. 2021;82(5):338–43. 10.1007/s00056-021-00289-z. 33765156

[13] The World Health Organization Quality of Life assessment (WHOQOL). position paper from the World Health Organization. Soc Sci Med. 1995;41(10):1403–9. 10.1016/0277-9536(95)00112-k. 8560308

[14] Cunningham SJ, Garratt AM, Hunt NP. Development of a condition-specific quality of life measure for patients with dentofacial deformity: I. Reliability of the instrument. Community Dent Oral Epidemiol. 2000;28(3):195–201. 10.1034/j.1600-0528.2000.280305.x. 10830646

[15] Jung MH. Quality of Life and Self-Esteem of Female Orthognathic Surgery Patients. J Oral Maxillofac Surg. 2016;74(6):1240. 10.1016/j.joms.2016.01.046. .e1-7. 26917200

[16] Al-Bitar ZB, Al-Ahmad HT. Anxiety and post-traumatic stress symptoms in orthognathic surgery patients. Eur J Orthod. 2017;39(1):92–7. 10.1093/ejo/cjw029. 27076465

[17] Zhou D, Wang LK, Wu HY, Gao L, Yang XD. Early-stage postoperative depression and anxiety following orthognathic surgery: a cross-sectional study. BMC Anesthesiol. 2024;24(1):338. 10.1186/s12871-024-02726-z. 39342085 PMC11438367

[18] Selvaraj AK, Benington PCM, Murphy L, Ayoub AF. Psychology input to an orthognathic clinic: Patients’ perception of service quality. Surgeon. 2019;17(6):340–5. 10.1016/j.surge.2018.11.001. 30661952

[19] Kashan DL, Horan MP, Wenzinger E, Kashan RS, Baur DA, Zins JE, et al. Identification of Body Dysmorphic Disorder in Patients Seeking Corrective Procedures From Oral and Maxillofacial Surgeons. J Craniofac Surg. 2021;32(3):970–3. 10.1097/SCS.0000000000007370. 33645953

[20] Qasim SS, Qasim RS, Alyamani AM. Recognizing and managing body dysmorphic disorder among patients seeking plastic surgery: a narrative review. Aesthetic Plast Surg. 2026;50(10):3758–65. 10.1007/s00266-026-05738-x. 41803311

[21] Koblenzer CS. Body dysmorphic disorder in the dermatology patient. Clin Dermatol. 2017;35:298–301. 10.1016/j.clindermatol.2017.01.002. 28511828

[22] Salık H, Uslu Ö, Şahin M. The experiences of patients enrolled in community mental health centers regarding the illness process: a phenomenological study. J Psychiatr Ment Health Nurs. 2025;32:288–96. 10.1111/jpm.13111. 39291391 PMC11891422

[23] Ahad AA, Sanchez-Gonzalez M, Junquera P. Understanding and addressing mental health stigma across cultures for improving psychiatric care: a narrative review. Cureus. 2023;15(5):e39549. 10.7759/cureus.39549. 37250612 PMC10220277

[24] Chen WL, Wei LC. Cross-cultural reflections on community mental health centres: lessons from Turkey for Taiwan and beyond. J Psychiatr Ment Health Nurs. 2025;32:1286–7. 10.1111/jpm.70020. 40832964

[25] Shelton AT, Houghton NY, Morris DO, Latchford GL, Bekker HL, Munyombwe T. The development and validation of a psychological questionnaire for patients undergoing orthognathic treatment. Orthod Craniofac Res. 2015;18(1):51–64. 10.1111/ocr.12061. 25418550

[26] Dons F, Mulier D, Maleux O, Shaheen E, Politis C. Body dysmorphic disorder (BDD) in the orthodontic and orthognathic setting: A systematic review. J Stomatol Oral Maxillofac Surg. 2022;123(4):e145–52. 10.1016/j.jormas.2021.10.015. 34728407

[27] Mueller SC, De Cuypere G, T’Sjoen G. Transgender Research in the 21st Century: A Selective Critical Review From a Neurocognitive Perspective. Am J Psychiatry. 2017;174(12):1155–62. 10.1176/appi.ajp.2017.17060626. 29050504

[28] Coleman E, Radix AE, Bouman WP, Brown GR, de Vries ALC, Deutsch MB, et al. Standards of care for the health of transgender and gender diverse people, version 8. Int J Transgend Health. 2022;23(sup1):S1S259. 10.1080/26895269.2022.2100644. PMC9553112 36238954

[29] Butterfield KJ, Marks PL, McLean L, Newton J. Quality of Life Assessment After Maxillomandibular Advancement Surgery for Obstructive Sleep Apnea. J Oral Maxillofac Surg. 2016;74(6):1228–37. 10.1016/j.joms.2016.01.043. 26917205

[30] Sarwer DB, Crerand CE. Body image and cosmetic medical treatments. Body Image. 2004;1(1):99–111. 10.1016/S1740-1445(03)00003-2. 18089144

[31] Sáenz-Ravello G, Carrasco García P, Fan S, Al-Nawas B. Prevalence of Body Dysmorphic Disorder in Patients Seeking Orthognathic Surgery: A Systematic Review and Meta-Analysis. J Oral Maxillofac Surg. 2025;83(11):1351–62. 10.1016/j.joms.2025.07.003. 40782826

[32] Pfeifer JH, Berkman ET. The Development of Self and Identity in Adolescence: Neural Evidence and Implications for a Value-Based Choice Perspective on Motivated Behavior. Child Dev Perspect. 2018;12(3):158–64. 10.1111/cdep.12279. 31363361 PMC6667174

[33] Xiao S, Shi L, Dong F, Zheng X, Xue Y, Zhang J, et al. The impact of chronic diseases on psychological distress among the older adults: the mediating and moderating role of activities of daily living and perceived social support. Aging Ment Health. 2022;26(9):1798–804. 10.1080/13607863.2021.1947965. 34238092

[34] Dong Y, Cheng L, Cao H. Impact of informal social support on the mental health of older adults. Front Public Health. 2024;12:1446246. 10.3389/fpubh.2024.1446246. 39391160 PMC11464432

[35] Srinath S, Jacob P, Sharma E, Gautam A. Clinical Practice Guidelines for Assessment of Children and Adolescents. Indian J Psychiatry. 2019;61(Suppl 2):158–75. 10.4103/psychiatry.IndianJPsychiatry_580_18. 30745694 PMC6345125

[36] Takatsuji H, Kobayashi T, Kojima T, Hasebe D, Izumi N, Saito I, et al. Effects of orthognathic surgery on psychological status of patients with jaw deformities. Int J Oral Maxillofac Surg. 2015;44(9):1125–30. 10.1016/j.ijom.2015.02.003. 26004311

[37] Baherimoghaddam T, Tabrizi R, Naseri N, Pouzesh A, Oshagh M, Torkan S. Assessment of the changes in quality of life of patients with class II and III deformities during and after orthodontic-surgical treatment. Int J Oral Maxillofac Surg. 2016;45(4):476–85. 10.1016/j.ijom.2015.10.019. 26603194

[38] Dalmeijer SWR, van Riet TCT, Ho JTF, Becking EAG. Evaluating Genioplasty Procedures: A Systematic Review and Roadmap for Future Investigations. Craniomaxillofac Trauma Reconstr. 2025;18(1):5. 10.3390/cmtr18010005. 40271466 PMC11995826

