# Supplementary material for: Attitudes and practices regarding preoperative psychological evaluation in orthognathic surgery: a scenario-based national survey of Turkish oral and maxillofacial surgeons
Source: BMC Oral Health. 2026 Jun 23;26:1660. doi: 10.1186/s12903-026-08928-w (PMC13540918; doi:10.1186/s12903-026-08928-w)
Supplement: Supplementary file 1 [file 12903_2026_8928_MOESM1_ESM.docx]

**Oral and Maxillofacial Surgeons' Perspectives on Preoperative**

**Psychological Assessment in Orthognathic Surgery Patients**

**Survey Form**

**Section 1: Demographic Information**

**1. What is your gender? ***

*Please select only one option.*

○ Female

○ Male

○ Prefer not to say

**2. Your age**

*(Open text)*

**3. What is your training status in oral and maxillofacial surgery? ***

*Please select only one option.*

○ I am currently in specialty / PhD training.

○ I have completed my specialty / PhD training.

○ I am a trainer.

**4. Where is your workplace located? ***

*Please select only one option.*

○ University / training hospital

○ Private hospital

○ Private clinic

○ Oral and dental health center

○ Other:

**5. How long have you been practicing your profession? ***

*Please select only one option.*

○ 0–5 years

○ 6–10 years

○ More than 10 years

**6. Which of the following best describes the number of orthognathic surgery patients you have evaluated in the past year?**

*Please select only one option.*

○ 1–10 patients

○ 11–25 patients

○ 26–50 patients

○ 51 or more patients

**Section 2: Psychological Evaluation Practices**

**7. Do you perform psychological evaluation in your orthognathic surgery patients? ***

*Please select only one option.*

○ Yes

○ No (Please proceed to Question 9)

**8. What tool do you use for psychological evaluation in your orthognathic surgery patients?**

*Please select only one option.*

○ A specifically developed scale

○ Interviews based on my personal experience

○ I refer the patient to a psychiatrist / psychologist

○ Other:

**8a. If you use a specifically developed scale for psychological evaluation in your orthognathic surgery patients, please specify.**

*(Open text)*

**Section 3: Referral Necessity and Clinical Assessment**

**9. Do you think that preoperative psychological evaluation is necessary for all orthognathic surgery patients?**

*Please select only one option.*

○ Yes

○ No

○ Undecided

**10. Approximately what percentage of orthognathic surgery patients in your clinic could benefit from psychological/psychiatric referral?**

*Please select only one option.*

○ 100%

○ 75%

○ 50%

○ 25%

○ 10%

○ None

○ I have no opinion

**11. Approximately what percentage of orthognathic surgery patients in your clinic are referred for psychological evaluation/treatment?**

*Please select only one option.*

○ 100%

○ 75%

○ 50%

○ 25%

○ 10%

○ None

○ I have no opinion

**Section 4: Question 12 — Referral Scenarios (Likert Scale)**

**Please evaluate the necessity of referral for preoperative psychological evaluation and support in orthognathic surgery patients in the following situations.**

**12a If the patient requests surgery despite not having a significant functional or aesthetic problem, referral for psychological evaluation and support is necessary.**

*Please select only one option.*

○ Strongly Disagree

○ Disagree

○ Undecided

○ Agree

○ Strongly Agree

**12b A history of psychiatric conditions (past or recent) requires referral for psychological evaluation and support.**

*Please select only one option.*

○ Strongly Disagree

○ Disagree

○ Undecided

○ Agree

○ Strongly Agree

**12c Seeking opinions from multiple doctors before making a decision requires referral for psychological evaluation and support.**

*Please select only one option.*

○ Strongly Disagree

○ Disagree

○ Undecided

○ Agree

○ Strongly Agree

**12d Patients showing signs of body dysmorphic disorder should be referred for psychological evaluation and support.**

*Please select only one option.*

○ Strongly Disagree

○ Disagree

○ Undecided

○ Agree

○ Strongly Agree

**12e Patients with significant social anxiety or low self-esteem should be referred for psychological evaluation and support.**

*Please select only one option.*

○ Strongly Disagree

○ Disagree

○ Undecided

○ Agree

○ Strongly Agree

**12f Having unrealistic expectations requires referral for psychological evaluation and support.**

*Please select only one option.*

○ Strongly Disagree

○ Disagree

○ Undecided

○ Agree

○ Strongly Agree

**12g Recently developed concerns about orthognathic surgery require referral for psychological evaluation and support.**

*Please select only one option.*

○ Strongly Disagree

○ Disagree

○ Undecided

○ Agree

○ Strongly Agree

**12h If the clinician senses a psychological risk factor based on previous clinical experience, referral for psychological evaluation and support is necessary.**

*Please select only one option.*

○ Strongly Disagree

○ Disagree

○ Undecided

○ Agree

○ Strongly Agree

**12i Communication or compliance problems experienced with the patient during previous treatment processes require referral for psychological evaluation and support.**

*Please select only one option.*

○ Strongly Disagree

○ Disagree

○ Undecided

○ Agree

○ Strongly Agree

**12j Family pressure or objection regarding orthognathic surgery requires referral for psychological evaluation and support.**

*Please select only one option.*

○ Strongly Disagree

○ Disagree

○ Undecided

○ Agree

○ Strongly Agree

**12k In cases where the patient is considered insufficient in coping with the stress of the surgical process, referral for psychological evaluation and support is necessary.**

*Please select only one option.*

○ Strongly Disagree

○ Disagree

○ Undecided

○ Agree

○ Strongly Agree

**Section 5: Question 13 — Assessment by Patient Groups (Matrix)**

**Please evaluate the necessity of preoperative psychological assessment in the following patient groups.**

**13a. Demographic Factors ***

*Please select only one option in each row.*

|  | **1 - Definitely Not Necessary** | **2 - Not Necessary** | **3 - Undecided** | **4 - Necessary** | **5 - Definitely Necessary** |
| --- | --- | --- | --- | --- | --- |
| Patients with ongoing growth and development | ○ | ○ | ○ | ○ | ○ |
| Young patients (16–25 years) | ○ | ○ | ○ | ○ | ○ |
| Older patients | ○ | ○ | ○ | ○ | ○ |

**13b. Deformity and Type of Surgery ***

*Please select only one option in each row.*

|  | **1 - Definitely Not Necessary** | **2 - Not Necessary** | **3 - Undecided** | **4 - Necessary** | **5 - Definitely Necessary** |
| --- | --- | --- | --- | --- | --- |
| Borderline skeletal Class III cases | ○ | ○ | ○ | ○ | ○ |
| Genioplasty cases | ○ | ○ | ○ | ○ | ○ |
| Skeletal Class III cases | ○ | ○ | ○ | ○ | ○ |
| Skeletal Class II cases | ○ | ○ | ○ | ○ | ○ |

**13c. Medical and Surgical History-Related Factors ***

*Please select only one option in each row.*

|  | **1 - Definitely Not Necessary** | **2 - Not Necessary** | **3 - Undecided** | **4 - Necessary** | **5 - Definitely Necessary** |
| --- | --- | --- | --- | --- | --- |
| Patients with obstructive sleep apnea | ○ | ○ | ○ | ○ | ○ |
| Patients undergoing gender transition | ○ | ○ | ○ | ○ | ○ |
| Patients who previously underwent orthognathic or aesthetic surgery and are planned for revision surgery | ○ | ○ | ○ | ○ | ○ |
| Patients with cleft lip/palate | ○ | ○ | ○ | ○ | ○ |

**Section 6: Question 14 — Barriers**

**14. What is the main factor that prevents you most from performing preoperative psychological evaluation or referring orthognathic surgery patients for this purpose?**

*Please select only one option.*

○ Not knowing reliable scales / questionnaires that can be used as part of psychological evaluation

○ Not knowing a psychiatrist / psychologist to refer to

○ It takes too much time

○ Cost of referral

○ Not being sure whom to refer to

○ The experience that feedback obtained after psychological evaluation does not contribute to the treatment process

○ Fear that the patient may react negatively or refuse to see a psychologist / psychiatrist

○ Believing that psychological evaluation is not routinely necessary

○ Believing that referral would be more appropriate postoperatively when necessary

○ Other:

** Indicates mandatory question*
