# Supplementary material for: Attitudes and practices regarding preoperative psychological evaluation in orthognathic surgery: a scenario-based national survey of Turkish oral and maxillofacial surgeons
Source: BMC Oral Health. 2026 Jun 23;26:1660. doi: 10.1186/s12903-026-08928-w (PMC13540918; doi:10.1186/s12903-026-08928-w)
Supplement: Supplementary file 2 [file 12903_2026_8928_MOESM2_ESM.docx]

**Checklist for Reporting Results of Internet E-Surveys (CHERRIES)**

| ***Checklist Item*** | ***Explanation*** | ***Page Number*** |
| --- | --- | --- |
| Describe survey design | A cross-sectional, scenario-based online survey was conducted among oral and maxillofacial surgeons in Türkiye. The study used a convenience sampling approach based on voluntary participation. | Lines 105-107 |
| IRB approval | The study was approved by the Ethics Committee of Lokman Hekim University (Protocol No: 2025066). | (Lines 149–150) |
| Informed consent | Participants were informed about the study through an invitation email distributed via the Association of Oral and Maxillofacial Surgery Society (AÇBİD). The invitation included detailed information regarding the purpose of the study, the identity of the investigators, the voluntary nature of participation, and the confidentiality of responses.  The email stated that participation was entirely voluntary, that no personally identifiable information would be collected, and that responses would be used solely for scientific purposes. Participants were also informed that they could decline participation or discontinue the survey at any time without any consequences.  The estimated completion time of the survey (5–10 minutes) was clearly indicated.  Completion of the questionnaire following access via the provided survey link was considered as implied informed consent. | (Lines 109–110, Supplementary File 1) |
| Data protection | No personally identifiable data were collected. All responses were recorded anonymously using a secure online platform and were accessible only to the research team. | (Lines 132–133) |
| Development and testing | The questionnaire was developed based on a review of the relevant literature and expert input from oral and maxillofacial surgeons. Although a formal pilot study was not conducted, the survey items were reviewed and refined to ensure clarity, relevance, and clinical applicability. Prior to distribution, the usability and technical functionality of the electronic questionnaire (Google Forms) were tested to ensure proper operation, accessibility, and data recording. | (Lines 105–109) |
| Open survey versus closed survey | This was a closed survey distributed via the Association of Oral and Maxillofacial Surgery Society (AÇBİD) mailing list to a defined group of oral and maxillofacial surgeons.  Access to the survey was provided through a web link included in the invitation email. While the link could be accessed by recipients, the survey was not publicly advertised or openly available to the general public. | (Lines 109–110) |
| Contact mode | Initial contact with participants was made via the Internet through the official AÇBİD mailing list. | (Lines 109–110) |
| Advertising the survey | The survey was distributed via the AÇBİD email communication system. The invitation included a brief description of the study aim and encouraged voluntary participation. | (Lines 109–110) |
| Web/E-mail | The survey was conducted using an online platform (Google Forms), and responses were automatically recorded in a database. | (Lines 105–107) |
| Context | The survey was distributed via the official mailing list of the Association of Oral and Maxillofacial Surgery Society (AÇBİD), a national professional organization representing oral and maxillofacial surgeons in Türkiye. The mailing list is primarily used for professional communication, including academic announcements, clinical discussions, and educational activities. Its members consist mainly of practicing oral and maxillofacial surgeons working in academic institutions, training hospitals, and private practice settings. As the survey was distributed through a professional network, the sample was limited to individuals actively engaged in the field. However, participation was voluntary, and surgeons with a particular interest in psychosocial aspects of care may have been more likely to respond, potentially introducing self-selection bias. |  |
| Mandatory/voluntary | Participation was entirely voluntary. | (Lines 132–133) |
| Incentives | No incentives were offered. |  |
| Time/Date | Data were collected between June 1 and July 1, 2025. | (Lines 133–134) |
| Randomization of items or questionnaires | No randomization or alternation of questionnaire items was applied; all participants were presented with the same sequence of questions. |  |
| Adaptive questioning | Limited adaptive questioning (skip logic) was applied, allowing certain question to be skipped based on previous response. |  |
| Number of Items | The questionnaire was presented on a single page and consisted of 27 items in total, including 11 main questions with additional sub-items. |  |
| Number of screens (pages) | The questionnaire was presented on a single page, and participants accessed all items by scrolling vertically. |  |
| Completeness check | The survey platform (Google Forms) was configured to require responses for key sociodemographic variables (gender, level of training, workplace, and professional experience), preventing submission of incomplete entries for these items. For all questionnaire items, participants were required to select only one response option, ensuring consistency in data entry and preventing multiple selections. Other questionnaire items were optional; however, all participants provided complete responses, and no missing data were observed in the final dataset. No automated consistency checks were applied beyond mandatory field settings. |  |
| Review step | Participants were able to review and modify their responses before submitting the questionnaire. |  |
| Unique site visitor | Unique site visitors were not tracked, as the survey was distributed via a direct invitation link without requiring login or registration. No IP or cookie-based restrictions were applied to prevent multiple submissions; however, the survey was shared within a professional mailing list, which likely reduced the risk of duplicate responses. |  |
| View rate (Ratio of unique survey visitors/unique site visitors) | Not applicable. |  |
| Participation rate (Ratio of unique visitors who agreed to participate/unique first survey page visitors) | The response rate was 8.4%, based on approximately 702 invited participants and 59 completed responses. |  |
| Completion rate (Ratio of users who finished the survey/users who agreed to participate) | The questionnaire was presented on a single page without a separate consent or entry page; therefore, attrition during the survey process could not be assessed. All submitted questionnaires were complete, and no partially completed responses were recorded. |  |
| Cookies used | No cookies were used. |  |
| IP check | IP addresses were not used to identify duplicate entries. |  |
| Log file analysis | No log file analysis was performed. |  |
| Registration | The survey did not require user registration. |  |
| Handling of incomplete questionnaires | Only fully completed questionnaires were included in the analysis. Questionnaires that were not submitted (i.e., terminated before completion) were not recorded by the survey platform and were therefore not available for analysis.  Given the voluntary nature of participation, non-submission was considered equivalent to withdrawal from the survey. |  |
| Questionnaires submitted with an atypical timestamp | No time-based criteria were applied to exclude questionnaires. The time required to complete the survey was not recorded, and no cut-off values were defined for identifying atypical response times. |  |
| Statistical correction | No statistical weighting or adjustment methods (such as propensity score analysis) were applied. Given the exploratory nature of the study and the use of a convenience sample, the results were interpreted descriptively without attempts to statistically correct for sampling bias. |  |

This checklist has been modified from Eysenbach G. Improving the quality of Web surveys: the Checklist for Reporting Results of Internet E-Surveys (CHERRIES). J Med Internet Res. 2004 Sep 29;6(3):e34 [erratum in J Med Internet Res. 2012; 14(1): e8.]. Article available at [https://www.jmir.org/2004/3/e34](https://www.jmir.org/2004/3/e34/)/; erratum available <https://www.jmir.org/2012/1/e8/>. Copyright ©Gunther Eysenbach. Originally published in the [Journal of Medical Internet](http://www.jmir.org) Research, 29.9.2004 and 04.01.2012.

This is an open-access article distributed under the terms of the Creative Commons Attribution License (<https://creativecommons.org/licenses/by/2.0/>), which permits unrestricted use, distribution, and reproduction in any medium, provided the original work, first published in the Journal of Medical Internet Research, is properly cited.
