# Supplementary material for: Attitudes and practices regarding preoperative psychological evaluation in orthognathic surgery: a scenario-based national survey of Turkish oral and maxillofacial surgeons
Source: BMC Oral Health. 2026 Jun 23;26:1660. doi: 10.1186/s12903-026-08928-w (PMC13540918; doi:10.1186/s12903-026-08928-w)
Supplement: Supplementary file 3 [file 12903_2026_8928_MOESM3_ESM.docx]

**Supplementary File 3 . Comparison of the Analytic Sample and Respondents Excluded Due to “No Opinion” Responses**

| **Variable** | **Analytic Sample**  **(n=41)** | **Excluded Respondents**  **(n=18)** |
| --- | --- | --- |
| **Gender**  Female | 25 (61.0%) | 10 (55.6%) |
| Male | 16 (39.0%) | 8 (44.4%) |
| **Years of Professional Experience** |  |  |
| 0–5 years of experience | 13 (31.7%) | 11 (61.1%) |
| 6-10 years of experience | 12 (**29.3%)** | 5 (**27.8%)** |
| >10 years of experience | 16 (**39.0%)** | 2 (**11.1%)** |
| **Level of Training** |  |  |
| Currently in residency/PhD training | 10 (24.4%) | 10 (55.6%) |
| Completed residency/PhD training | 12 (**29.3%)** | 6 (**33.3%)** |
| Trainer/faculty member | 19 (**46.3%)** | 2 (**11.1%)** |
| **Number of Orthognathic Patients Evaluated in the Last Year** |  |  |
| 1–10 orthognathic cases/year | 15 (36.6%) | 13 (72.2%) |
| 11–25 orthognathic cases/year | 14 (**34.1%)** | 4 (**22.2%)** |
| 26–50 orthognathic cases/year | 9 (**22.0%)** | 0 (**0%)** |
| >50 orthognathic cases/year | 3 (**7.3%)** | 1 (**5.6%)** |
